# Supplementary material for: Leaf defenses of subtropical deciduous and evergreen trees to varying intensities of herbivory
Source: PeerJ. 2023 Nov 7;11:e16350. doi: 10.7717/peerj.16350 (PMC10637251; doi:10.7717/peerj.16350)
Supplement: Supplemental Information 1 — The data represent mean values (standard error). [file peerj-11-16350-s001.docx]

## Supplemental Materials

**Table S1.** Stand-level information for fourteen dominant tree species at a large experimental subtropical forest plot in the Guanshan National Nature Reserve, China. The data represent mean values (standard error).

| Number | Species | Family | Life span | Mean DBH (cm) | Mean Height  (m) | Shade tolerance | Habit |
| --- | --- | --- | --- | --- | --- | --- | --- |
| 1 | *Liquidambar formosana* | Hamamelidaceae | deciduous | 21.6 (0.9) | 17.0 (0.8) | intolerant | tolerant |
| 2 | *Padus buergeriana* | Rosaceae | deciduous | 14.7 (0.8) | 15.8 (0.8) | intolerant | tolerant |
| 3 | *Quercus acutissima* | Fagaceae | deciduous | 25.3 (0.9) | 16.4 (1.0) | intolerant | tolerant |
| 4 | *Alniphyllum fortunei* | Styracaceae | deciduous | 18.4 (0.9) | 15.8 (0.8) | intolerant | pioneer |
| 5 | *Acer wilsonii* | Aceraceae | deciduous | 10.3 (0.8) | 9.8 (0.7) | neutral | tolerant |
| 6 | *Sapium discolor* | Euphorbiaceae | deciduous | 11.9 (0.5) | 12.5 (1.3) | intolerant | tolerant |
| 7 | *Choerospondias axillaris* | Anacardiaceae | deciduous | 24.1 (0.9) | 14.9 (0.7) | intolerant | pioneer |
| 8 | *Elaeocarpus japonicus* | Elaeocarpaceae | evergreen | 15.1 (0.8) | 17.0 (0.7) | neutral | tolerant |
| 9 | *Daphniphyllum oldhamii* | Daphniphyllaceae | evergreen | 14.3 (0.5) | 14.2 (0.5) | tolerant | tolerant |
| 10 | *Rhododendron latoucheae* | Ericaceae | evergreen | 10.5 (0.5) | 9.2 (0.6) | tolerant | tolerant |
| 11 | *Machilus thunbergii* | Lauraceae | evergreen | 11.3 (0.5) | 11.2 (1.1) | tolerant | tolerant |
| 12 | *Schima superba* | Theaceae | evergreen | 13.8 (0.6) | 9.8 (0.8) | intolerant | pioneer |
| 13 | *Castanopsis tibetana* | Fagaceae | evergreen | 15.2 (0.4) | 14.0 (0.5) | tolerant | tolerant |
| 14 | *Castanopsis carlesii* | Fagaceae | evergreen | 12.5 (0.5) | 11.7 (1.1) | tolerant | tolerant |

**Table S2. The comparison of traits between deciduous and evergreen subtropical tree species.** Abbreviations: carbon (C), nitrogen (N), phosphorus (P), non-structural carbohydrates (NSC). Significantly higher values are shown in bold. The data represent mean values for deciduous and evergreen leaves. Significant differences between deciduous and evergreen species are denoted with **P*<0.05, ***P*<0.01, ****P* < 0.001.

| Traits | Mean | | t | Sig. (2-tailed) |
| --- | --- | --- | --- | --- |
|  | deciduous | evergreen |  |  |
| IV (Importance Value) | 2.81 | 2.79 | -0.053 | 0.958 |
| C (%) | 55.28 | 56.80 | 1.698 | 0.094 |
| N (%) | **2.22** | 1.39 | -7.140 | <0.001^***^ |
| P (g/kg) | **1.40** | 1.01 | -5.140 | <0.001^***^ |
| NSC (%) | **11.78** | 9.51 | -3.225 | 0.002^***^ |
| Tannin (%) | 1.37 | 1.50 | 0.792 | 0.431 |
| Lignin (%) | 23.67 | 24.45 | 0.918 | 0.362 |
| Cellulose (%) | 21.99 | 21.20 | -0.882 | 0.381 |
| Hemicellulose (%) | 18.64 | **19.53** | 2.133 | 0.037^*^ |
| C/N | 26.52 | **43.08** | 7.017 | <0.001^***^ |
| N/P | 16.31 | 14.60 | -1.456 | 0.150 |
| N/Lignin | **0.10** | 0.06 | -6.679 | <0.001^***^ |
| NSC/Lignin | **0.51** | 0.39 | -3.776 | <0.001^***^ |
| NSC/Cellulose | **0.55** | 0.45 | -3.124 | 0.003^***^ |
